# A meta-analysis on the lifetime and period prevalence of self-injury among adolescents with depression

**DOI:** 10.3389/fpubh.2024.1434958

**Published:** 2024-07-31

**Authors:** Yanli Wu, Yanhong Zhang, Chengzhen Wang, Beibei Huang

**Affiliations:** ^1^Emergency Department, The Affiliated Brain Hospital of Nanjing Medical University, Nanjing, Jiangsu, China; ^2^Nursing Department, The Affiliated Brain Hospital of Nanjing Medical University, Nanjing, Jiangsu, China

**Keywords:** adolescent, non-suicidal self-injury, self-injury, depression, meta-analysis

## Abstract

**Background:**

Adolescents are vulnerable to mental disorders due to physiological, psychosocial, and cognitive changes during this critical developmental stage. Depression, in particular, can lead to high-risk behaviors such as self-injury (SI) and suicide. This study aims to estimate the pooled prevalence of SI behaviors among adolescents with depression.

**Materials and methods:**

We systematically searched databases including EMBASE, Scopus, PubMed, and Web of Sciences for relevant articles published on adolescents with depression from January 1, 2000, to January 1, 2024. The quality assessment of the studies was conducted using the Joanna Briggs Institute (JBI) criteria. The global prevalence of SI was calculated based on a random effects model using Stata software version 17.

**Results:**

Our analysis included 29 studies involving 12,934 adolescents. The lifetime prevalence of SI was 52% (95% Confidence Interval [CI]: 41–64), while the period prevalence was 57% (95% CI: 49–64). Notably, a significant relationship was observed between the prevalence of SI and the year of publication of articles (*p* = 0.002). Furthermore, publication bias was not significant for both lifetime prevalence (*p* = 0.281) and period prevalence (0.358).

**Conclusion:**

The prevalence of self-injurious behaviors in adolescents with depression is alarmingly high, with more than half of adolescents having engaged in these high-risk behaviors during their lifetime or within the last year. Given the associated risk of suicide, it is crucial to identify individuals at risk and provide timely interventions.

## Introduction

Self-injury (SI) or non-suicidal self-injury (NSSI) refers to the direct and intentional destruction of body tissues without suicidal intent (1). Common forms of SI include cutting, burning, scratching and hitting oneself (2). The concept of these behaviors was first discussed in the book “Man Against Himself,” where self-injury was described as a form of “partial suicide” by the author (3). Despite continuous research efforts, there is still no generally accepted definition for this behavior. Various terms, such as “syndrome of delicate self-cutting,” “deliberate self-harm,” “self-wounding,” and “moderate self-mutilation” have been proposed, which has made it difficult to understand these behaviors due to the diversity and lack of consensus (4–7).

Adolescence refers to the transitional stage between the ages of 10 and 19. Although this stage is typically considered problem-free, nearly 20% of young people experience a mental health problem (8). The prevalence of SI in the general population is 4% (9), while 15% of teenagers and young adults have had a history of SI (10). Additionally, 46% of ninth and tenth grade students have engaged in at least one SI behavior during the past year (11).

SI results from dysregulation of emotions, internalizing behaviors, and inability to cope effectively. The results of studies conducted on different populations show that people who harm themselves have higher levels of depression and anxiety (12–15). Individuals with depressive symptoms often use SI as a coping mechanism for negative emotions, providing temporary relief followed by subsequent feelings of sadness and guilt (16). If SI is performed repeatedly, it fails to alleviate depressive symptoms and instead exacerbates negative emotions over time, increasing the risk of depression (17).

SI is distinct from suicide, but they can be connected. Suicidal behavior involves any action with the intention of ending one’s own life. Self-injury behaviors do not aim to cause death but serve as coping mechanisms for emotional pain, stress, or trauma and occur more frequently than suicide attempts (18). The primary risk of SI is its potential to become chronic and progress into other types of self-injurious acts, such as suicide attempts (19). Despite the distinction between these behaviors and suicide attempts, which typically do not require immediate medical attention or result in death, such dangerous behaviors may serve as a precursor to suicidal behaviors in the future (20, 21). Experts argue that despite differences in intent, epidemiology, and lethality between SI and suicide, these two behaviors often co-occur (21–23).

Numerous studies have been undertaken to investigate the prevalence of SI among depressed individuals, yielding mixed results. Additionally, only a limited portion of these studies have focused on adolescents. This study aims to answer the question: what is the period prevalence (the proportion with the characteristic at any point during a specified time period) and lifetime prevalence (the proportion who have ever had the characteristic at some point in their lives) of self-injury among depressed adolescents? Thus, this study was conducted with the objective of estimating the period and lifetime prevalence of SI among depressed adolescents.

## Materials and methods

This meta-analysis was conducted with the aim of estimating the pooled prevalence of SI among depressed adolescents on published articles from 2000 to 2024 based on the Preferred Reporting Items for Systematic Review and Meta-analyses PRISMA checklist (24).

### Search strategy

In this review, Medline, Web of Science, Scopus, and EMBASE electronic databases were searched from January 2000 to January 2024 with the keywords “self-injurious behavior,” “self-mutilation,” “self-harm,” “depression,” “depressive disorder,” “adolescent,” and their combinations. Google Scholar and ResearchGate were searched to access other related studies. Searching and screening of articles were conducted from January to March 2024. Initially, abstracts and titles of retrieved articles were screened to determine eligibility. Irrelevant papers were removed, and the full text of the remaining articles was reviewed by two authors independently. The references of the articles were manually searched to access additional relevant studies.

### Eligibility criteria

The inclusion criteria were: Publication in English between 2000 and 2024, observational studies involving depressed adolescents (people aged 10 to 19), and reporting necessary data such as frequency or prevalence of SI. Studies conducted on other age groups, reviews, qualitative articles, editorials, and intervention were excluded from the analysis.

### Data extraction

Data were extracted by a member of the research team based on a pre-prepared form and then double-checked by a second reviewer. Any disagreements were resolved through discussion with a third author. The extracted information included the first author, year of publication, sample size, country, and frequency or prevalence of SI.

### Risk of bias assessment

The quality of the selected articles was evaluated independently by two authors. The Joanna Briggs Institute (JBI) critical appraisal checklist for prevalence studies was used to evaluate bias in selected articles. The JBI appraisal checklist comprises nine items, with answers of yes (score 1) and no, unclear and not applicable (score zero). The overall score ranges from 0 to 9, where a higher score indicates higher quality (25).

### Statistical analysis

The data was analyzed with Stata version 17 software. Due to the expected heterogeneity caused by differences in methodological approaches and geographic locations, the cumulative prevalence of SI was calculated using a random effects model (26). Heterogeneity was assessed using Cochran’s Q test (*X^2^*) and I^2^. I^2^ was evaluated with thresholds greater than or equal to 25%, greater than or equal to 50%, and greater than or equal to 75%, indicated low, medium, and high heterogeneity, respectively (27). A forest plot was used to display the estimated cumulative prevalence of SI (with 95% confidence interval [CI]). Sensitivity analysis was conducted to evaluate the robustness of the results, and meta-regression was performed to examine the relationship between the prevalence of SI and sample size, year of publication and mean age of participants. Subgroup analysis was performed to estimate the lifetime and period prevalence by the study countries (China and other countries). Publication bias was assessed using a funnel plot and Egger’s test.

## Results

### Selection results and study characteristics

In the initial search, 10,032 articles were retrieved. Of these, 4,767 were duplicate articles. After reviewing the title and abstract of the remaining articles, 4,722 were removed. The full text of 543 articles was then reviewed. At this stage, 18 review articles, 87 clinical trials, 12 articles with insufficient data, and 397 irrelevant articles were excluded. Finally, 29 studies published in English, with a sample size of 12,934 subjects, were analyzed.

The smallest and largest sample sizes were 44 (28) and 2,343 (29), respectively. Most studies were published in 2023 (*n* = 11) and 2022 (*n* = 8). Also, 76% of selected studies were conducted in China. The rest of the studies were related to the countries of Colombia (*n* = 1), Turkey (*n* = 1), United States (*n* = 1), England (*n* = 1), and South Korea (*n* = 3). In terms of quality, 24 studies had good quality and five studies had moderate quality. The mean age of participants was reported in 14 studies (Table 1).

**Table 1:** Characteristics of the included studies.

Author(s), Year	Sample size	Age	Country	Instrument	Depression diagnosis criteria	Quality	Prevalence	
							Lifetime	Period
Seong et al. (30)	85	12–18	South Korea	Single-item measure	DSM-IV	9	–	67.0
Hu et al. (31)	1969	10–17	China	DSHI	CES-DC	9	44.34	–
	2064	10–17	China	DSHI	CES-DC	9	–	53.44
Jiang et al. (32)	126	12–18	China	ANSSIQ	DSM-IV	9	–	66.66
Weng et al. (33)	391	–	China	OSI	SCL-90	6	–	60.87
Xiao et al. (34)	505	14.54	China	Single-item measure	DSM-IV	7	–	77.8
Shen et al. (35)	1,101	14.7	China	C-FASM	ICD-10	9	–	51.5
Ma et al. (36)	124	14.4	China	OSI	M.I.N.I.KID 5.0	8	42.8	–
Wang et al. (37)	562	–	China	NSSI-Q	ICD-11	8	–	59.25
Chang and Zhang (38)	366	–	China	Single-item measure	DSM-IV	6	78.96	–
Zhang et al. (29)	2,343	14.99	China	C-FASM	DSM-IV	9	–	76.06
Xie et al. (39)	501	19.03	China	ANSAQ	DSM-IV	9	79.44	–
Hua et al. (40)	224	15.3	China	CRSNSSI	DSM-IV	8	–	65.18
Hub et al. (41)	95	–	China	CRSNSSI	DSM-IV	6	–	49.47
Cao et al. (42)	225	11–19	China	BIS	DSM-IV	9	–	56.44
Jia et al. (43)	120	–	China	OSI	HAMD-17	6	50.0	–
Kang et al. (44)	116	16.0	South Korea	CBCL	DSM-IV	7	–	55.2
Song et al. (45)	505	14.54	China	Single-item measure	DSM-IV	9	–	77.82
Yi et al. (46)	120	18.81	China	OSI	DSM-IV	9	45.9	–
Ma et al. (47)	80	15.0	China	FASM	DSM-IV	9	–	50.0
Jiao et al. (48)	357	11–16	China	C-FASM	DSM-IV	7	–	42.3
Taş Torun et al. (49)	67	–	Turkey	ISAS	BDI	7	64.1	–
Qian et al. (50)	114	17.28	China	Single-item measure	DSM-IV	8	49.12	–
Huang and Liu (51)	53	–	China	BIS-11	DSM-IV	6	56.60	–
Huang et al. (52)	67	13–19	China	Single-item measure	DSM-IV	7	–	46.26
Valencia et al. (28)	44	12.84	Colombia	SHQ	CDI	7	20.4	–
Kim (53)	113	12–18	South Korea	K-SADS-PL	CDI	7	42.5	–
Asarnow et al. (54)	334	15.9	USA	K-SADS-PL	DSM-IV	8	–	23.9
Wilkinson et al. (55)	163	14.3	England	K-SADS-PL	DSM-IV	7	–	37

ISAS, inventory of statements about self-injury; DSHI, Deliberate Self-Harm Inventory; ANSSIQ, Adolescent Non-Suicidal Self-Injury Questionnaire; OSI, Ottawa Self-Injury Inventory; BNSSI-AT, The Brief Non-Suicidal Self-Injury Assessment Tool; C-FASM, Chinese version of functional assessment of self-mutilation; NSSI-Q, Non-Suicidal Self-Injury Questionnaire; ANSAQ, Adolescent Non-suicidal Self-injury Assessment Questionnaire; CRSNSSI, Clinician-Rated Severity of Non-suicidal Self-Injury; BIS, Body Investment Scale; CBCL, Child Behavior Checklist; K-SADS-PL, Kiddie-Sads-Present and Lifetime Version; FASM, functional assessment of self-mutilation; BIS-11, Adolescents Self-Harm Scale and Barratt Impulsivity Scale; SHQ, self-harm questionnaire. DSM-5, The Diagnostic and Statistical Manual of Mental Disorders, Fifth Edition; SDS, self-rating depression scale; ESCID, Early Warning System and Comprehensive Intervention for Depression; CDI, Children's Depression Inventory; ICD-10, International Classification of Diseases; Tenth Revision, ICD-11, International Classification of Diseases 11th Revision; BDI, Beck Depression Inventory; HAMD-17, Hamilton Rating Scale for Depression, 17 items; M.I.N.I.KID 5.0, Mini-International Neuropsychiatric Interview for Children and Adolescents; CES-DC, Center for Epidemiological Studies. Depression Scale for Children, DASS-21, Depression, Anxiety and Stress Scale - 21 Items; SCL-90, Symptom Checklist-90. Depressed adolescents are those who have been diagnosed based on the diagnostic or screening tools described in this table.

### Lifetime and period prevalence

**Figure 1:**
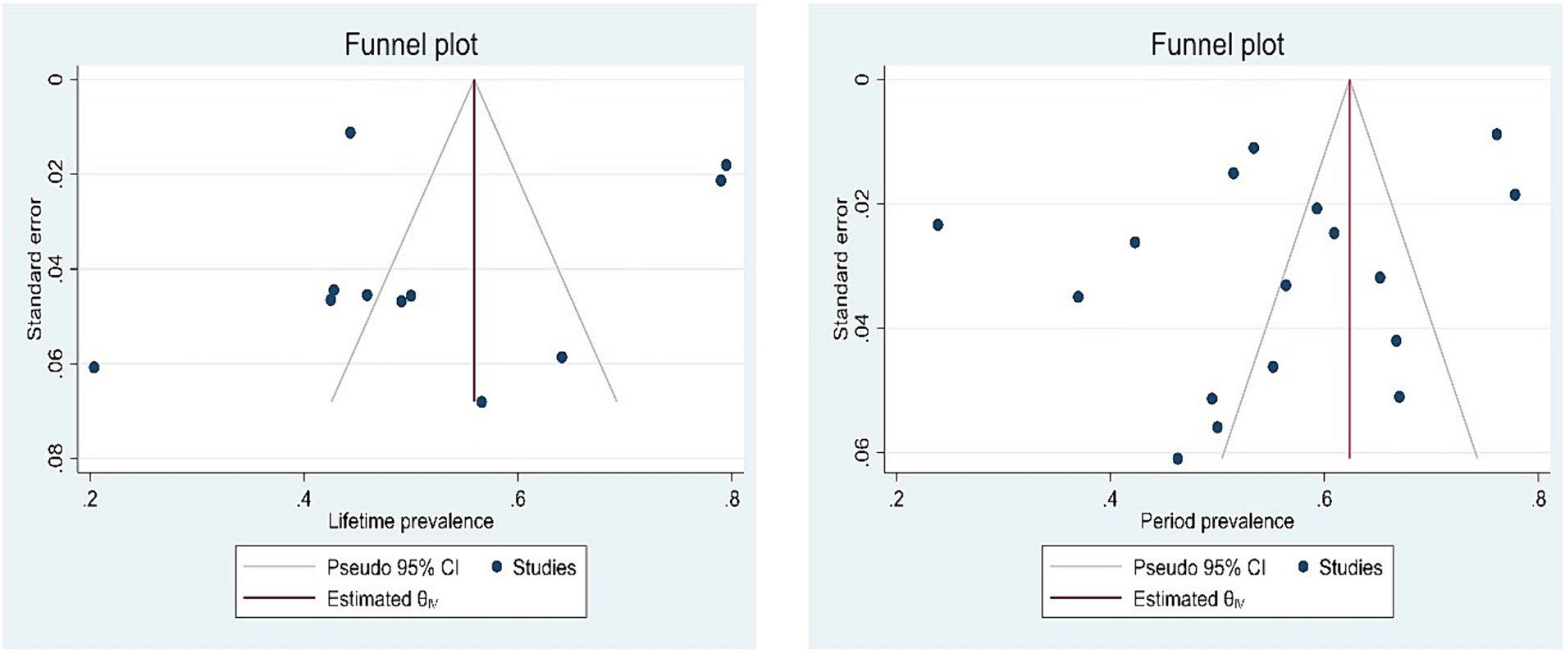
Flow chart of literature search.

Of the 29 analyzed studies, 10 reported lifetime prevalence (28, 36, 38, 39, 43, 46, 49–51, 53), and 18 (29–35, 37, 40–42, 44, 45, 47, 48, 52, 54, 55) reported period prevalence. In one study, both lifetime and period prevalence were reported (31). In this study, ‘period prevalence’ referred to the prevalence in the last 6 to 12 months. Specifically, six studies reported prevalence in the last 6 months (31, 33, 42, 44, 52, 54), while 12 studies reported prevalence in the last year (29, 30, 32, 34, 35, 37, 40, 41, 45, 47, 48, 55). The lowest and highest lifetime prevalence rates were 20.4% (28) and 78.9% (38), respectively. Similarly, the lowest and highest period prevalence rates were 23.9% (54) and 77.8% (45), respectively (Figure 1).

**Figure 2:**
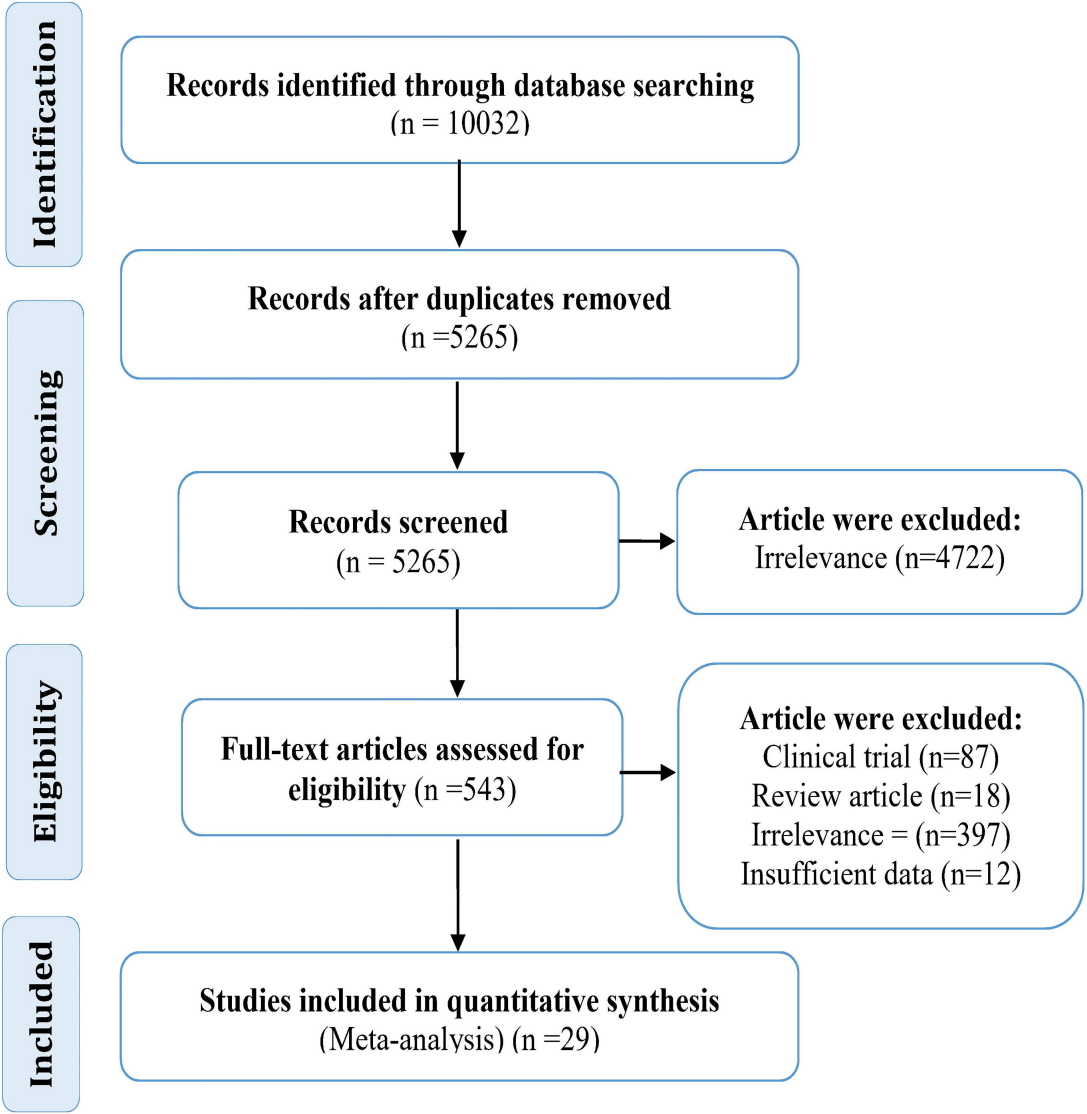
Forest plot of the lifetime and period prevalence of self-injury (SI) in adolescents.

**Figure 3:**
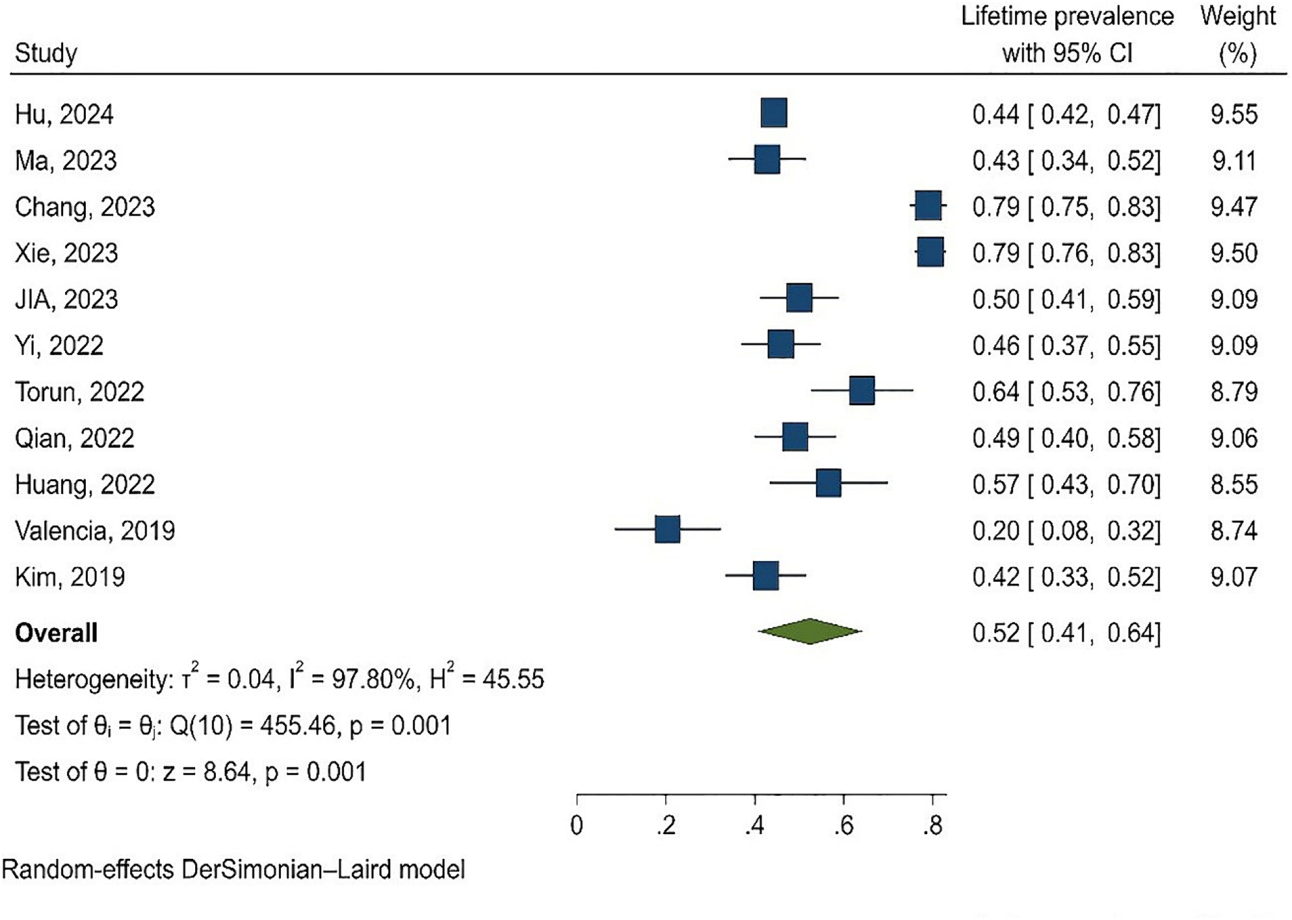
Forest plot of the period prevalence of self-injury (SI) in adolescents.

The results of the meta-analysis showed that the lifetime prevalence and period prevalence of SI were 52% (95% CI: 41–64, I2 = 97.80%) and 57% (95% CI: 49–64, I2 = 98.14%), respectively (Figures 2, 3). Since most of the analyzed studies were related to China, subgroup analysis was performed and lifetime and period prevalence were assessed separately by region (China and other countries). The findings revealed that lifetime prevalence in China was 56% (95% CI: 45–67) compared to 42% (95% CI: 18–67) in other countries. Similarly, period prevalence in China was 60% (95% CI: 54–66) versus 45% (95% CI: 27–64) in other countries. The period and lifetime prevalence of suicide in China and other countries were not significantly different.

The results of subgroup analysis by year showed that the lifetime prevalence of SI was 59% (95% CI: 43–76, I^2^ = 98.66%) in 2023 and 2024, and 46% (95% CI: 35–58, I^2^ = 86.51%) in previous years (Q = 1.60, *p* = 0.206). Additionally, the prevalence of SI in the years 2023 and 2024 was 62% (95% CI: 57–68, I^2^ = 95.95%), which was significantly higher than in previous years (47% with 95% CI: 35–60, I^2^ = 96.73; Q = 4.25, *p* = 0.039).

### Meta-regression analysis

Meta-regression was employed to investigate the relationship between lifetime and period prevalence with the year of publication of articles, mean age of participants and sample size. The results indicated a relationship between the period prevalence of SI and the year of publication of the articles (*p* = 0.002); such that between 2011 and 2024, the period prevalence of SI increased. In other words, from the publication of the oldest to the newest selected article, the period prevalence of SI has increased significantly.

### Publication bias

**Figure 4:**
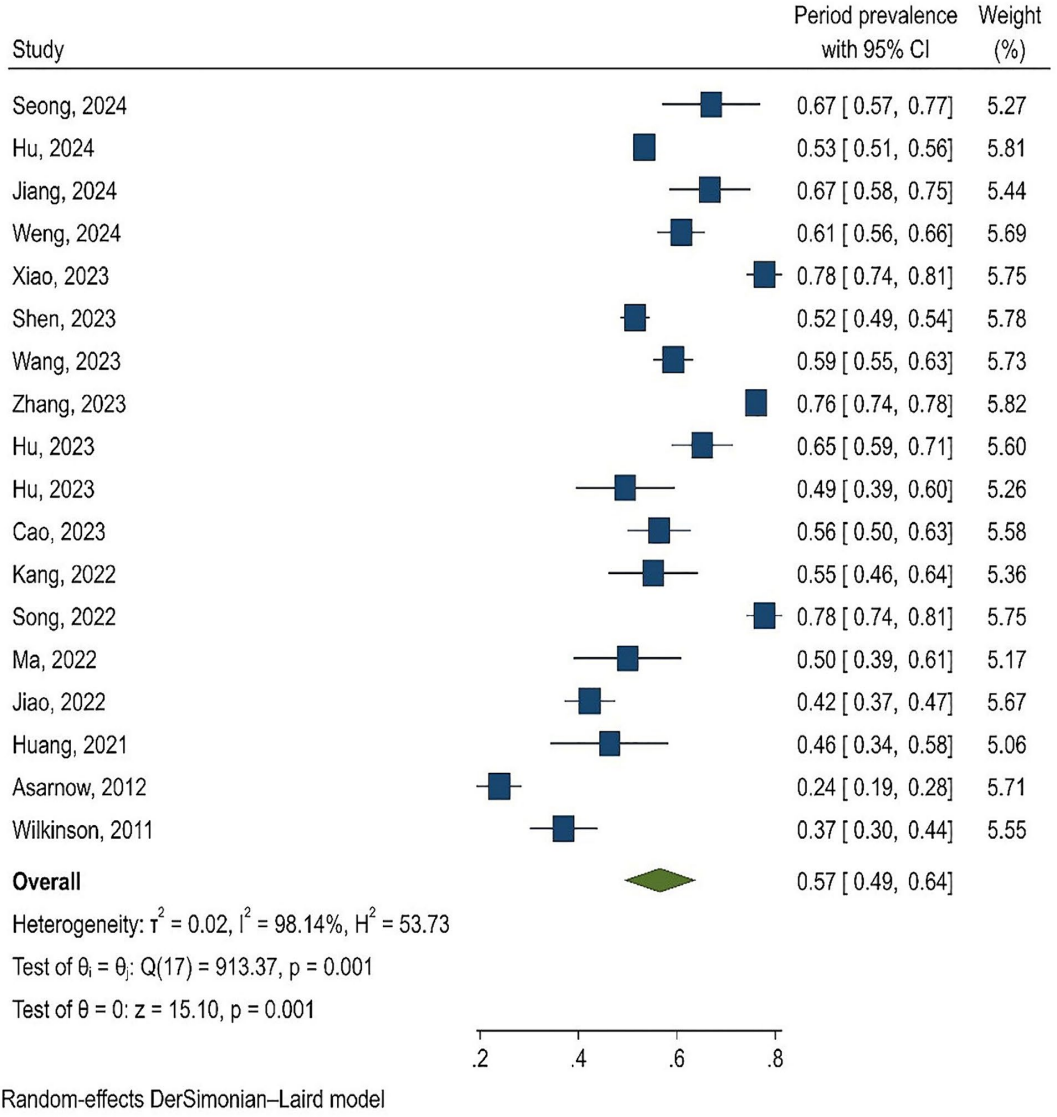
The results publication bias.

Publication bias was evaluated using Egger’s test and funnel plot. Publication bias was not significant in lifetime prevalence (*p* = 0.281) and period prevalence (0.358; Figure 4).

## Discussion

In this study, the lifetime and period prevalence of self-injurious behavior among adolescents with depression was systematically reviewed. The results of this study showed that the lifetime and period prevalence of SI among adolescents with depression were 52 and 57%, respectively. In other words, more than half of adolescents with depression have a history of self-injurious behaviors. Self-injury, in which a person overtly harms themselves, is important because of the difficulties in determining whether an adolescent is attempting to die (56). Research on adolescents who intentionally hurt themselves and seek treatment at hospitals shows that their actions are often impulsive. These incidents are usually triggered by family or friend issues, school difficulties, or disciplinary problems. Additionally, some cases are related to depression, anxiety, or behavioral disorders (57). The results of our study showed that the lifetime and period prevalence of SI among adolescents with depression were 52 and 57%, respectively. In other words, more than half of adolescents with depression have a history of self-injurious behaviors. Adolescence is a sensitive and vulnerable age in which a person learns the methods of internalizing and externalizing emotions, and if they learn and use unhealthy coping mechanisms, a wide range of problematic behaviors can appear (58).

Xiao et al.’s meta-analysis revealed that the prevalence of lifetime and period SI in non-clinical adolescents was 22 and 23.2%, respectively. However, our present study found a higher prevalence. One possible explanation for this discrepancy is the coexistence of depression with these behaviors, which may intensify the desire to engage in self-injury and suicidal thoughts (59). Based on the comparison of these studies, it can be concluded that although the prevalence of self-injury behaviors is high among adolescents, these behaviors are much higher among those who also suffer from depression.

People with depression may perceive life in a negative and distorted way through the activation of negative events. They think based on the “cognitive triad” that they are worthless, the world is unfair, and their future is hopeless. Triggered by this negative triad, adolescents with depressive disorder may self-harm to end their symptoms (60). The results of Zhang et al.’s study (2023) the prevalence of SI in the last year among 2,343 adults with depression was 76.06% (29). In another study that was conducted on 1,095 students, the frequency of self-injury among depressed students was 35.14% (61). It appears that the prevalence of self-injurious behaviors among depressed youth is lower than among adults who suffer from depression. The presence of depression can heighten the prevalence of suicidal ideation (SI) behaviors and increase the likelihood of suicide (62). Therefore, the most basic method of suicide prevention is the screening of suicidal behaviors. The results of Park et al.’s study showed that the prevalence of SI among depressed people was higher than non-depressed people (63). According to the Third Variable theory, a third variable must exist to connect SI to suicide. This variable can include psychological issues such as depression, suicidal thoughts, personality disorders, low self-esteem, and lack of family support (21, 64). The relationship between depression and SI is rarely linear, and a combination of predisposing factors such as genetics and previous experiences and acceleration of stressful events is involved in its occurrence (65). In another study, teenagers with SI behaviors had less suicidal thoughts and depression symptoms, more self-esteem, and more parental support compared to teenagers who attempted suicide (66). The results of a systematic review showed that SI behaviors with the following characteristics are associated with a significant rate of suicide attempts: duration of more than 1 year, use of multiple methods, self-cutting, frequent SI episodes, absence of physical pain during self-injury, severe physical harm, strong conscious intent to die, and concealment of the act (64).

Meta-regression results showed that from 2011 to 2024, the period prevalence of SI has increased significantly. The reason for this rising prevalence of SI may be the escalating level of daily stress and serious crises in recent years. The reason behind this finding may be that self-harm is associated with less stigma than many other inappropriate behaviors. Consequently, teenagers engage in self-harm more readily and even share their experiences with their peers. Further exploration of this issue necessitates qualitative studies.

Subgroup analysis revealed that while the lifetime and period prevalence of SI was higher in studies conducted in China compared to studies in other countries, this difference did not reach statistical significance. The prevalence of deliberate self-injury in 2023 and 2024 was significantly higher than in previous years. This increase may be attributed to heightened mental health concerns related to the onset of World War III and conflicts in the Middle East.

To the best of our knowledge, this meta-analysis is the first to examine the prevalence of self-injurious behaviors among depressed adolescents. SI behaviors are known as one of the predictors of suicide, so knowing the prevalence of SI in this age group provides new insights for researchers and policy makers to make decisions and adopt appropriate policies. This meta-analysis had several limitations: (1) only articles published in English were analyzed, (2) there were no other systematic review and meta-analysis studies conducted on adolescents, to which the findings of the present study could be compared, (3) Due to the small number of selected studies, studies with a small sample size were also included in the analysis, which may affect the reported estimated pooled prevalence.

## Conclusion

The prevalence of self-injurious behaviors among adolescents with depression is high, so that 57% of adolescents have committed these behaviors during the last year and 52% of adolescents have committed these behaviors during their lifetime. Considering that these behaviors can increase the chance of suicide, it seems necessary to identify people at risk.

## Data availability statement

The original contributions presented in the study are included in the article/supplementary material, further inquiries can be directed to the corresponding author.

## Author contributions

YW: Data curation, Methodology, Software, Writing – original draft, Writing – review & editing. YZ: Data curation, Formal analysis, Resources, Writing – original draft, Writing – review & editing. CW: Data curation, Methodology, Software, Writing – original draft, Writing – review & editing. BH: Investigation, Resources, Supervision, Validation, Writing – original draft, Writing – review & editing.
